# *Trypanosoma cruzi* Induces B Cells That Regulate the CD4^+^ T Cell Response

**DOI:** 10.3389/fcimb.2021.789373

**Published:** 2022-01-05

**Authors:** Martín Somoza, Adriano Bertelli, Cecilia A. Pratto, Ramiro E. Verdun, Oscar Campetella, Juan Mucci

**Affiliations:** ^1^ Instituto de Investigaciones Biotecnológicas, Universidad Nacional de San Martín-CONICET, Buenos Aires, Argentina; ^2^ Sylvester Comprehensive Cancer Center and Division of Hematology, Department of Medicine, University of Miami Miller School of Medicine, Miami, FL, United States

**Keywords:** B cell proliferation, Chagas disease, IL-21, regulatory B cells, T CD4^+^ cells

## Abstract

*Trypanosoma cruzi* infection induces a polyclonal B cell proliferative response characterized by maturation to plasma cells, excessive generation of germinal centers, and secretion of parasite-unrelated antibodies. Although traditionally reduced to the humoral response, several infectious and non-infectious models revealed that B lymphocytes could regulate and play crucial roles in cellular responses. Here, we analyze the trypomastigote-induced effect on B cells, their effects on CD4^+^ T cells, and their correlation with *in vivo* findings. The trypomastigotes were able to induce the proliferation and the production of IL-10 or IL-6 of naïve B cells in co-culture experiments. Also, we found that IL-10-producing B220^lo^ cells were elicited *in vivo*. We also found up-regulated expression of FasL and PD-L1, proteins involved in apoptosis induction and inhibition of TCR signaling, and of BAFF and APRIL mRNAs, two B-cell growth factors. Interestingly, it was observed that IL-21, which plays a critical role in regulatory B cell differentiation, was significantly increased in B220^+^/IL-21^+^ in *in vivo* infections. This is striking since the secretion of IL-21 is associated with T helper follicular cells. Furthermore, trypomastigote-stimulated B-cell conditioned medium dramatically reduced the proliferation and increased the apoptotic rate on CD3/CD28 activated CD4^+^ T cells, suggesting the development of effective regulatory B cells. In this condition, CD4^+^ T cells showed a marked decrease in proliferation and viability with marginal IL-2 or IFNγ secretion, which is counterproductive with an efficient immune response against *T. cruzi*. Altogether, our results show that B lymphocytes stimulated with trypomastigotes adopt a particular phenotype that exerts a strong regulation of this T cell compartment by inducing apoptosis, arresting cell division, and affecting the developing of a proinflammatory response.

## Introduction

B cells are the main effectors of the humoral immunity, which is mediated by their activation and secretion of different classes of immunoglobulins against the original antigen (Nutt et al., 2015) However, these cells exhibit other antibody-independent functions such as their performance as antigen-presenting-cell (APC) or cytokine secretion that modulate the entire immune response (Shen and Fillatreau, 2015; Getahun and Cambier, 2019).

The immune response associated with survival against infection by *Trypanosoma cruzi*, the agent of Chagas disease, infection relies on the generation of a CD4^+^ T helper 1 (Th1) profile and a strong CD8^+^ T response that is, however, unable to resolve the infection (Tarleton et al., 2000; Kumar and Tarleton, 2001). Although the T cell response is the one that carries out the effector action, several other cell types such as macrophages and dendritic cells are also involved and have been well studied (Acevedo et al., 2018). On the other hand, beyond the particular parasite-induced proliferative effects that have been observed, our knowledge on the effect of *T. cruzi* infection on B cells is still unclear. *T. cruzi* induces polyclonal lymphocytes proliferation and unspecific hypergammaglobulinemia during the early stage of the acute phase of the infection with an antigen-specific delayed response (Ortiz-Ortiz et al., 1980; Minoprio et al., 1988; Rottenberg et al., 1993; el Bouhdidi et al., 1994; Bermejo et al., 2011). This unspecific antibody response is characterized by their maturation as plasma cells and the secretion of parasite-unrelated antibodies, a response that is maintained all along the infectious process (D’Imperio Lima et al., 1986; Minoprio et al., 1988; Bermejo et al., 2011). This pathogenic manifestation is known as polyclonal expansion and is histologically characterized by an excessive generation of germinal centers (typical and atypical) accompanied by extensive generation of extra-follicular B cell response (Montes et al., 2007; Bermejo et al., 2011). Particularly, different cell extracts or antigen preparations from either epimastigotes (the insect replicative stage) or trypomastigotes (the mammal infective stage) induce a T cell-independent B cell proliferative response and even several components of the parasite are related to this effect, although just a few are molecularly identified such as *trans*-sialidase, malate or glutamate dehydrogenase and proline racemase (Montes et al., 1999; Reina-San-Martín et al., 2000; Montes et al., 2002; Gao et al., 2002; Montes et al., 2006). In contrast, studies with live trypomastigotes are scarce.

In several models, both infectious and non-infectious, the importance of B cells in the development of the T cell response has been addressed and it has been shown that B lymphocytes can act as regulators of the T response and play an important role in the effector activities of the cellular response (Candando et al., 2014; Gorosito Serrán et al., 2015; Lykken et al., 2015; Catalán et al., 2021). There is indirect evidence of the regulatory role of B cells in the development of the immune response against *T. cruzi*. Although there are some discrepancies, most of the evidence supports the idea that both, partial (Xid mouse) and absolute (μMT mouse) deficiencies in B cells generate important outcomes in the development of the infection, generally associated with a stronger inflammatory response presenting higher levels of IFNγ, IL-6, IL-18 and TNFα in serum (Minoprio et al., 1993; Cardillo et al., 2007; Gorosito Serrán et al., 2017; da Rocha et al., 2019). In the μMT mouse absolute depletion model, Gorosito Serrán et al. demonstrate that the increased of infected animals mortality was associated with an unconventional CD4^+^ T cell exacerbated response leading to an uncontrolled immune response and increased inflammation (Gorosito Serrán et al., 2017). Furthermore, it was reported that B cells help to develop the CD8^+^ response in mice infected with *T. cruzi* (Fiocca Vernengo et al., 2020). These results lead us to infer that B cells undoubtedly play a regulatory role in the overall immune response against the parasite. However, little is known about the mechanisms associated with this ability, and there is almost no direct cellular evidence that mechanistically interprets and supports these findings.

In this work, we analyze the effects of live trypomastigotes on B cells and, in turn, their regulation of the T cell compartment. In search for clues that could provide evidence, we followed the proliferative response of B cells induced by trypomastigotes, and we then analyzed their effects on T cells and their correlation with the *in vivo* findings.

## Materials and Methods

### Mice

The protocol of this study was approved by the Committee on the Ethics of Animal Experiments of the Universidad Nacional de San Martín (UNSAM), following the recommendations of the Guide for the Care and Use of Laboratory Animals of the National Institutes of Health. C57BL/6J mice were obtained from The Jackson Laboratory and bred in our facilities. Male mice (60 to 90 days old) were used in all experiments.

### Parasites and Infections

CL Brener parasites were obtained from infected Vero cell culture supernatants. For trypomastigotes purification, supernatants were centrifuged at high speed and incubated for 4h at 37°C and 5% CO_2_, allowing motile parasites to swim towards the top of the suspension. Then, the trypomastigote-enriched upper layer was removed carefully.

For mice infections, RA trypomastigotes were obtained from infected mice whole blood and 50 parasites were injected by intraperitoneal route (i.p.). Animals were euthanized at day +14.

### T and B Naïve Cell Purification

Mouse splenocytes were obtained as described previously (Díaz et al., 2015). For CD4^+^ T cell purification, MojoSort Mouse CD4 Naïve Cell Untouched Isolation kit was used, following the manufacturer’s instructions (Biolegend). Cell purity (>90%) was checked by flow cytometry. For CD43^-^ naïve B cell purification, Dynabeads Mouse CD43 (Untouched B Cells) was used, following the manufacturer’s instructions (Thermo Fisher Scientific) Cell purity (>95%) was checked by flow cytometry.

### Cell Cultures

Purified naive cells were plated at 1,5x10^6^/ml in the presence of 1x10^6^/ml trypomastigotes for 3-5 days depending upon the experiment. Stimulation with 5μg/ml anti-CD40 (1C10 clone) was used. For T cell stimulation assays, purified naive T cells were plated at 1x10^6^/ml in CD3 (1μg/ml) and CD28 (2μg/ml) pre-coated plates and incubated for 3 days. In B-T co culture experiments, cells were plated at a 1:1 ratio.

### ELISAs

For cytokine secretion assays, cell culture supernatants were collected at corresponding time points, depending on the experiment. The cytokine concentrations in culture supernatants were assayed by ELISA using paired antibodies (Biolegend). For IgG assays, plates were first coated with anti-total mouse Ig’s and then incubated with anti-IgG specific antibody, both from Sigma.

Standard curves were performed in the case of cytokines. For total IgG, OD values were normalized to the control condition.

Briefly, plates were coated with the corresponding antibody in Phosphate Buffer (pH 9) ON at 4°C. Blocking was performed using TBS-BSA 1% solution for 1h at RT. Samples were then incubated for 1h at RT. Detection biotinylated antibodies were incubated in TBS-BSA 0.1% solution for 1h at RT. Streptavidin-HRP conjugate (Biolegend) was added for 30’ at RT in the dark. Washing steps were performed with TBS-Tween 20 0.05% three times each. Colorimetric reaction was performed with TMB (Sigma) and hydrogen peroxide in 10mM Citrate Buffer (pH 5.5). Finally, reaction was halted with Sulfuric Acid 0.2M and absorbance at 450nm was measured in Filtermax F5 (Molecular Devices).

### RNA Isolation and RT-qPCR

Cells were pelleted and lysed in TRIzol solution (Thermo Fisher Scientific). mRNA was obtained following manufacturer’s instructions and treated with RQ1 DNAse (Promega) to eliminate possible genomic DNA contamination. RNA was quantified with NanoDrop spectrophotometer ND-1000 (Thermo). Then, RNA was retro-transcripted to cDNA with M-MLV reverse transcriptase (Promega) using Oligo dT primers (Integrated DNA Technologies), according to manufacturer’s recommendations.

For qPCR, Kapa SYBR^®^ Fast qPCR kit (Kapa Biosystems) was used and reactions were followed in Gene Amp 7500 Sequence Detection System (Applied Biosystems).

Polymerase chain reaction was performed using the following primer pairs: BAFF Fwd: 5’-AAGACCGTTTTCTCCAGTCCTTT-3’, Rv: 5’-CATGTGTCACCCAAGGCAAA-3’; APRIL Fwd: 5’-CTCTTGGCCACCTCACTTCTG-3’, Rv: 5’-GGAGATGAGGCTGGCATGA-3’. As housekeeping control genes: Ubiquitin C Fwd: 5’- GCCCAGTGTTACCACCAAGA-3’, Rv: 5’-CCCATCACACCCAAGAACA-3’; GAPDH Fwd: 5’-CATCACTGCCACCCAGAAGACTG-3’, Rv: 5’-ATGCCAGTGAGCTTCCCGTTCAG-3’. Samples were analyzed by triplicate. Analysis of data was performed with LinReg software.

### Flow Cytometry Analysis

Cell suspensions were incubated in PBS-azide plus anti-FcR monoclonal antibody (CD16/32; Clone 93) for 30 min on ice. Then, anti-CD80 (Clone 16-10AI), anti-CD86 (Clone PO3), anti-MHC-II (CloneM5/114.15.2), anti-CD11c (Clone N418), or anti-CD11b (Clone M1/70) mAb, anti-B220 (Clone RA3-6B2), anti-CD1d (Clone 1B1), anti-CD5 (Clone 53-7.3), anti-CD21 (Clone Bu32), anti-CD23 (Clone B3B4), anti-FasL (Clone MFL3), anti-PDL1 (MIHS), anti-Syndecan-1 (Clone 281-2), anti-IL-6 (MP5-20F3), anti-IL-10 (JES5-16E3), anti-IL-21 (mhalx21) conjugated to distinct fluorochromes, were added in the recommended concentrations. After 1h, cells were washed, fixed with 1% *p-*formaldehyde in PBS, and analyzed by flow cytometry. All antibodies and their isotype controls were from Biolegend.

For B10 detection, splenocytes were harvested and cultured for 48h in the presence of anti-CD40 (1C10 clone) with PMA (50 ng/ml) and Ionomycin (500 ng/ml) (Sigma) for 6h and Monensin plus Brefeldin A (Biolegend) for 4h before staining. For intracellular staining, BD Cytofix/Cytoperm™ Fixation/Permeabilization Solution Kit (Becton Dickinson) was used, following the manufacturer’s instructions. Cells were analyzed in a CyFlow space (Partec, Germany) or FACSAriaII (Becton Dickinson) cytometers.

### Proliferation Dye Staining

Cell suspensions were stained with CFSE following the manufacturer’s instructions (Thermo Fisher Scientific). Briefly, cell suspensions were cultured in 5μM dye solution in PBS for 15min at 37°C. The reaction was halted adding 2% cold FBS and washed.

### Apoptosis Assays

Annexin V Apoptosis Detection Kit was used, following the manufacturer’s instructions (Thermo Fisher Scientific). Briefly, cells were incubated in a calcium-enriched buffer, which allows binding of Annexin-V to exposed phosphatidylserine in apoptotic cell membranes. Before the flow cytometer run, cells were incubated in a propidium iodide solution to check the membrane integrity.

### Statistical Analysis

Analysis of variance (ANOVA) with Bonferroni’s correction or Student’s *t* test were used for statistical analysis.

## Results

### *T. cruzi* Trypomastigotes Induce Proliferation and Activation of B Cells

**Figure 1:**
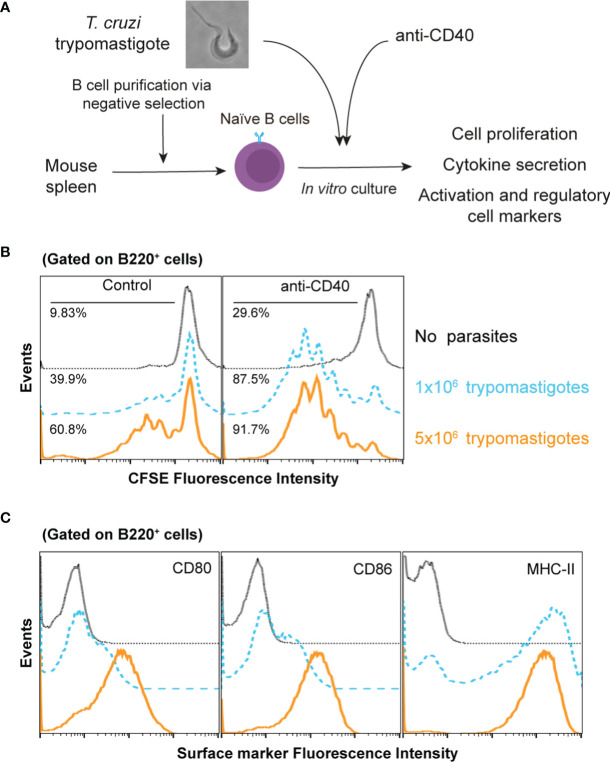
*T. cruzi* trypomastigotes induce naïve B cell proliferation and activation. **(A)** Schematic experimental representation **(B)** CFSE-stained naïve B cells were cultured in several conditions. Dotted lines: untreated B cells; dashed lines 1x10^6^ trypomastigotes/ml; solid lines 5x10^6^ trypomastigotes/ml. **(C)** CD80, CD86 and MHCII surface expression by naive B cells after culture with 1x10^6^ trypomastigotes/ml, as determined by flow cytometry. Dotted lines: isotypes control; dashed lines untreated B cells; solid lines cells cocultured with trypomastigotes.

It has been observed *in vivo* that unspecific B cells proliferate during *T. cruzi* infection, which could be considered to occur in a T-dependent and/or in a T-independent manner (D’Imperio Lima et al., 1985; Minoprio et al., 1987). To study this phenomenon, highly purified naïve B cells (**Supplementary Figure 1**) stained with CFSE were cocultured with *T. cruzi* trypomastigotes during 72h (**Figure 1A**). As shown in **Figure 1B**, live trypomastigotes induced a drastic increase in the proliferation of B cells *in vitro* in a dose dependent manner. Then, to analyze the possible influence of T-cells, a CD40 agonist antibody was added because the CD40-CD40L interaction is crucial to stimulate and maintain of B cell response (Cyster and Allen, 2019). We found that the B cell proliferation was strikingly enhanced when the agonist anti-CD40 antibody was added to the culture to mimic the presence of CD40L (**Figure 1B**). These findings support that T cells signaling plays a significant role in this process and that both T-independent and T-dependent activation are involved. In fact, in addition to an unrelated IgM response a strong collection of IgG antibodies is known to also occur.

Then, we wanted to determine the activation state of proliferating B cells exposed to trypomastigotes for 72h by determining the expression levels of the major histocompatibility complex (MHC) and costimulatory molecules. Analysis of these cell activation markers in these cultures indicated that trypomastigotes induced an activated state determined by the expression of MHC-II, CD80, and CD86 on the B cells surface (**Figure 1C**). The presence of these molecules ensures the interaction between the B cell and T cells allowing the development of their immune synapse. The immune synapse is a cornerstone in communication between the cells involved, improving the delivery efficiency of secreted factors, and allowing the interaction of surface molecules, resulting in the regulation of the immune response.

**Figure 2:**
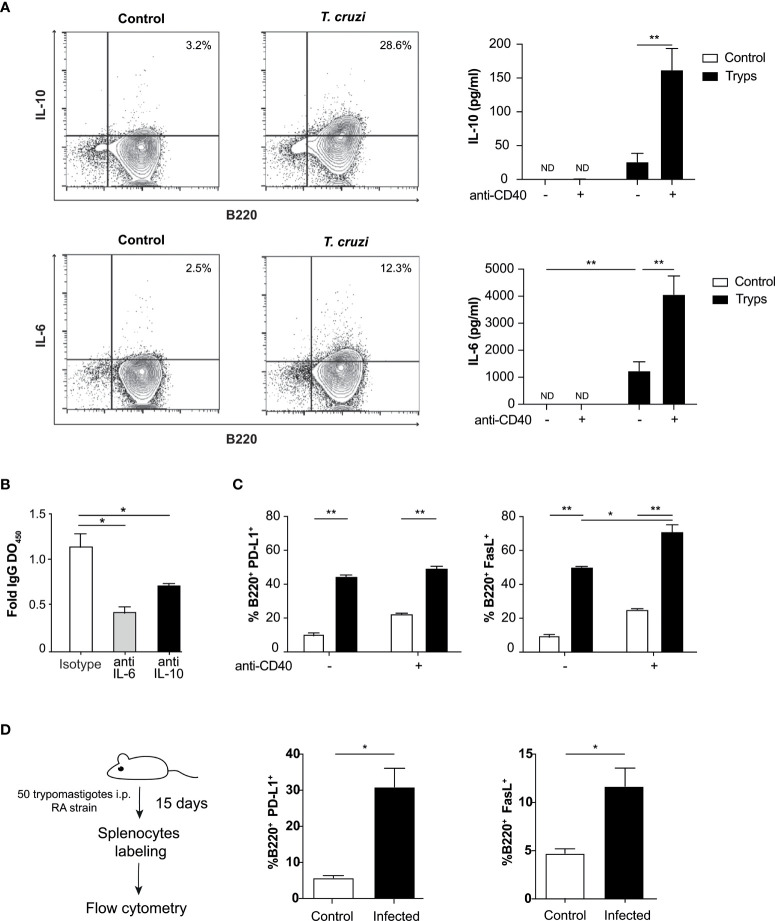
B cells secrete cytokines and express surface regulatory ligands after stimulation with *T. cruzi* trypomastigotes. **(A)** Detection of B cell derived cytokines by flow cytometry and ELISA upon parasite stimulation. Supernatants from naïve B cells cocultured with trypomastigotes and treated or not with anti-CD40 were tested by ELISA for IL-10 (top panel) and IL-6 (bottom panel). The presence of an agonist anti-CD40 antibody induced ILs even strongly. Control (unfilled bars): unstimulated B cells, trypomastigotes (filled histograms): cells stimulated by the addition of 1x106 trypomastigotes/ml. **p < 0.001. ND: not detected **(B)** B cells were cultured in presence of IL-6 or IL-10 blocking antibodies or an isotype control and total IgG OD were determined by ELISA. *p < 0.05 **(C)** B cells were cultured with 1x106 trypomastigotes/ml and the anti-CD40 antibody and surface expression of PD-L1 and Fas-L regulatory proteins were then tested by cytometry. Control (unfilled bars): unstimulated B cells, trypomastigotes (filled bars): cells stimulated by the addition of trypomastigotes. Two-way ANOVA with Bonferroni’s correction was used, *p < 0.05, **p < 0.001. **(D)** Surface expression of PD-L1 and FasL in splenocytes from naïve or *T. cruzi* infected mice evaluated by cytometry. Student’s t test was used, *p < 0.05.

Then, we sought to analyze the cytokine content of the supernatants coming from B cells/trypomastigotes co-cultures. We detected increased concentrations of IL-6 and IL-10 (**Figure 2A**), which are known to be associated with the regulation of the immune system (Couper et al., 2008; Jones and Jenkins, 2018). This increment was even more pronounced when an agonist anti-CD40 antibody was included to mimic the interaction with the corresponding T cells. No significant differences were detected for IL-2, IL-4, IL-17, or IFNγ (not shown) in these conditions. Intracellular staining analysis revealed the secretion of IL-10 and IL-6 only in B220^+^ cells (**Figure 2A**), showing that these cytokines were secreted from B cells and not from any other possible population of minor contaminant cells.

We then tested whether the proliferation induced by trypomastigotes was mediated by IL-6 and IL-10, which are also involved in the activation and proliferation of B cells (Tosato et al., 1988; Rousset et al., 1992; Kopf et al., 1994; Burdin et al., 1997). Surprisingly, when the association of these cytokines with autocrine B cell proliferation was tested by adding neutralizing monoclonal antibodies to block cytokine/receptor interactions, we found that they were not involved in the process (**Supplementary Figure 2**). As described above, during *T. cruzi* infection an unrelated IgM and IgG antibodies response is known to be elicited (D’Imperio Lima et al., 1986; Minoprio et al., 1988; Bermejo et al., 2011). Regarding that, we evaluated the IgG spontaneous secretion in these conditions. We found that in B lymphocytes stimulated with trypomastigotes and cultured with neutralizing antibodies to IL-6 or IL-10, IgG secretion decreased (**Figure 2B**), suggesting that these cytokines could be involved in the unspecific secretion of immunoglobulins.

Since we have found regulatory cytokines in the supernatants of our co-cultures, we determined the expression of well-known regulatory ligands on the surface of B cells. We found that *T. cruzi* trypomastigotes positively regulate the expression of FasL and PD-L1, which are involved in inducing apoptosis of target cells and inhibiting TCR signaling, respectively. The parasite *per se* could induce the expression of both regulatory molecules. However, only when the anti-CD40 antibody was included in the co-culture, an enhancement in FasL expression was observed. This enhancement of PD-L1 levels was not detected in the previous conditions (**Figure 2C**). Finally, we wondered if the expression of these two molecules is enhanced in B cells in our in vivo mouse infection model. Mice were infected with 50 trypomastigotes (RA strain), and on day 15 post-infection the percentages of B220^+^/PD-L1^+^ and B220^+^/FasL^+^ were evaluated. As shown in **Figure 2D**, we found that both populations were expanded significantly in the infected mice, six times for PD-L1 and twice for FasL. This results are in concordance with previous reports in mice and human (Zuñiga et al., 2002; Girard et al., 2021). The presence of these molecules on the surface of B cells allows them to elicit various regulatory mechanisms in addition to those mediated by cytokines.

**Figure 3:**
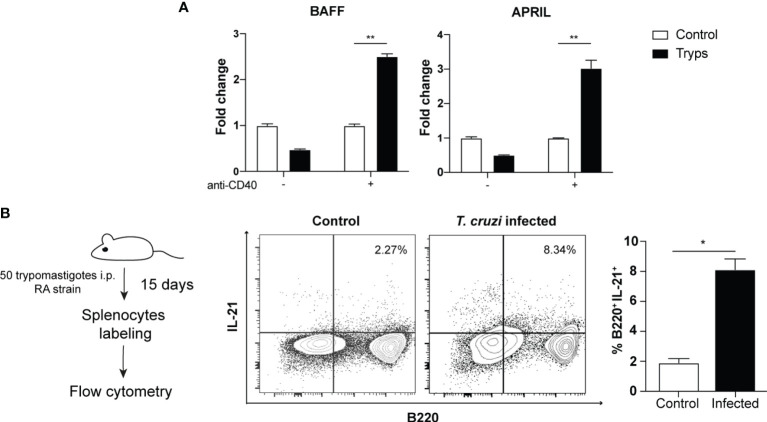
Trypomastigotes induce the expression of B cell growth and differentiation factors. **(A)** The mRNAs of BAFF and APRIL were quantified by qPCR after coculture of naïve B cells with trypomastigotes, in the presence or not of an agonist anti-CD40 antibody. Tryps (filled bars): cells stimulated by the addition of 1x10^6^ trypomastigotes/ml. Two-way ANOVA with Bonferroni’s correction was used, **p < 0.001. **(B)** Cytoplasmic expression of IL-21 in B220^+^/CD4^-^ cells harvested from naïve or infected mice. Student’s *t* test was used, *p < 0.05.

Next, we searched whether *T. cruzi* trypomastigotes can increase BAFF and APRIL transcripts, two well-known B cell growth factors involved in the B cell survival and/or differentiation (Hsu et al., 2002; Mackay et al., 2003). Consistent with the promotion of cell division, we found that both mRNAs were upregulated measured by qPCR only in the presence of anti-CD40 antibody (**Figure 3A**). This finding is relevant since it was previously described that these factors are mainly secreted by macrophages and dendritic cells and T cells (Mackay et al., 2003; Ng et al., 2005), but the absence of accessory cells in these cultures suggests that trypomastigotes act directly on B cells for their induction.

Several reports emphasize the relevance of IL-21 in the induction of IL-10 secreting regulatory B cells (Tedder, 2015), although it is also associated to proliferation/apoptosis, plasma cell differentiation or immunoglobulin production (Spolski and Leonard, 2014). Generally, the IL-21 secretion is associated mainly, although not exclusively, with the CD4^+^ follicular (Tfh) and CD4^+^ Th17 cells (Lüthje et al., 2012; Spolski and Leonard, 2014). Given the function attributed to this cytokine, we sought to determine whether B cells could be effectively secreting IL-21in the context of *T. cruzi* infection.

Based on the described above, we performed *in vivo* assays to validate this hypothesis in the mouse model. After 2 weeks of infection with 50 trypomastigotes of *T. cruzi* (RA strain), we detected an increase in IL-21 producing B220^+^ cells (**Figure 3B**). We found two different populations, one B220^lo^ and the other B220^high^ that secreted IL-21 during the acute stage of the infection. This finding is noteworthy as IL-21 expression has only been reported in Hodgkin’s lymphoma (B-cell-related lineage) but not in physiologically regulated or untransformed B cells (Scheeren et al., 2008).

Collectively, we have observed here that trypomastigotes can activate and induce proliferation and a regulatory phenotype in naive B cells, secreting cytokines involved in immune regulation and the induction of regulatory ligands on the cell surface.

### Trypomastigote-Stimulated B Cells Exert CD4^+^ T Cell Regulation

**Figure 4:**
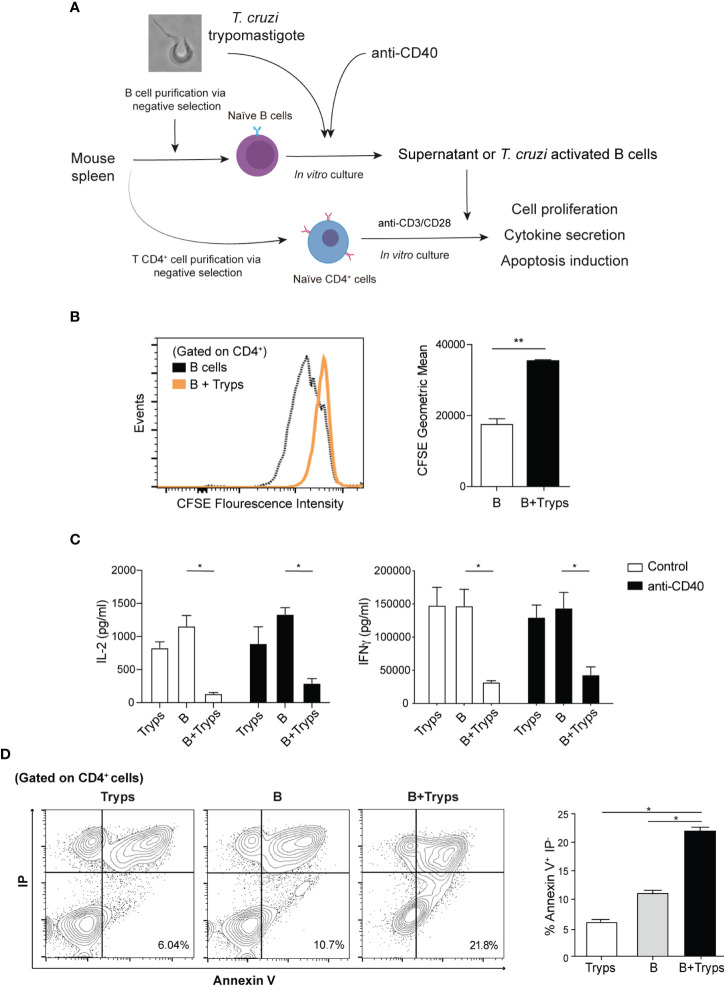
Supernatants from trypomastigote-stimulated B cells halted CD4^+^ T cells proliferation and altered the induced cytokine profile. **(A)** Schematic experimental representation. **(B)** CFSE proliferation labeling of CD4^+^ T cells after addition of conditioned media from B cell cultures. B cells; supernatants from B cells alone, B+Tryps: B cells plus trypomastigotes cocultures. Student’s *t* test was used, **p < 0.001. **(C)** Cytokine ELISA from CD3/CD28 stimulated CD4^+^ T cells in the presence of conditions media from diverse conditions. Tryps: supernatants from trypomastigotes alone, B: supernatants from B cells alone, B+Tryps: supernatants from B cells plus trypomastigotes cocultures. Two-way ANOVA with Bonferroni’s correction was used, **p < 0.001. **(D)** CD4^+^T cells stained by both propidium iodide and Annexin-V detected by flow cytometry. Tryps: CD4^+^ T cells cultured in the presence of conditioned media from trypomastigotes; B: CD4^+^ T cells cultured in the presence of conditioned media from unstimulated B cells; B+Tryps: CD4^+^ T cells cultured in the presence of conditioned media from B cells plus trypomastigotes. Student’s *t* test was used, **p < 0.001.

To test whether this B cells regulatory phenotype exerts functional activity on CD4^+^ T cells, the conditioned medium from trypomastigote-stimulated B cells was assayed on purified naïve CD4^+^ T cells pre-treated with antibodies anti-CD3 and CD28 (**Figure 4A**). As indicated by CFSE staining, the proliferation of CD4^+^ T cells was drastically dampened in these conditions (**Figure 4B**).

We then analyzed the supernatant cytokine content and determined that the secretion of IL-2, the main proliferative cytokine for CD4^+^ T cells clonal expansion, was significantly reduced (**Figure 4B**). Next, we analyzed whether the induced T helper profile was altered by measure of the released cytokine to the medium. Also we measured the IFNγ secretion, typically associated with Th1 profile, and we found a drastically reduction (**Figure 4C**). Note that the conditioned media from trypomastigotes cultured alone did not have any effect on CD4^+^ T cells, and that the regulation is only exerted when B cells interact with parasites (**Figure 4C**). Therefore, the conditioned media of the B cells stimulated with trypomastigotes exerted a strong regulation of the CD4^+^ T cells response because their cell division was inhibited, and the elicited cytokine secretion was altered.

Considering into account the observed upregulation of the regulatory ligands, the ability of B cells stimulated with trypomastigotes to induce apoptosis in CD4^+^ T cells was also tested. As expected, T cells cultured in the presence of parasites pre-stimulated B cells showed a significant increase in the apoptotic population (Annexin-V^+^/IP^-^ population) (**Figure 4D**), which correlates with the augmented number of hypodiploid TCD4^+^ cells when stimulated with B cells (**Supplementary Figure 3**). This result might constitute a regulatory mechanism to avoid the immune response against the parasite.

### Different IL-10 Producing B Cell Subsets Are Present in *T. cruzi*-Infected Mice

**Figure 5:**
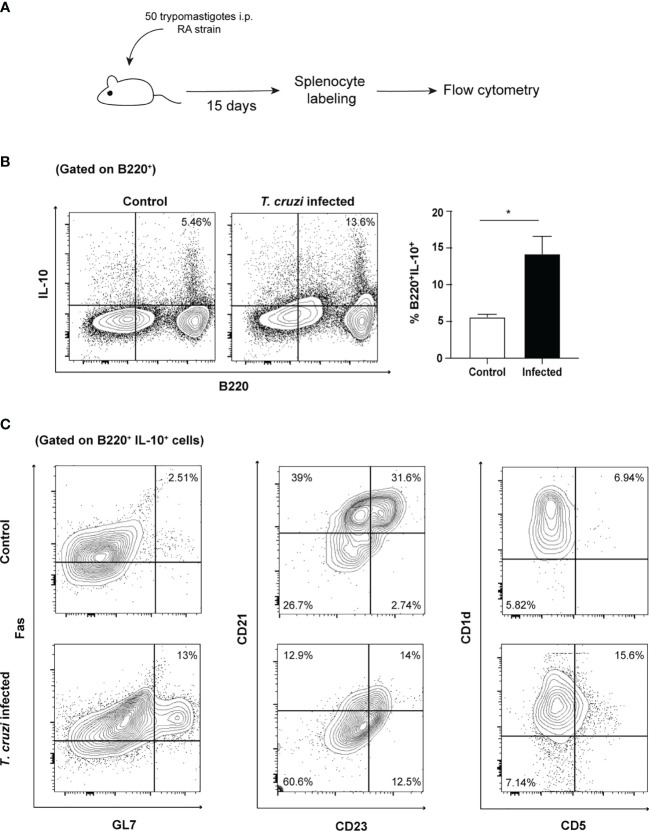
IL-10-producing B cells are expanded during *in vivo T. cruzi* infection and present several phenotypes. **(A)** Schematic experimental representation. **(B)** Cytoplasmic IL-10 presence in splenocytes from naïve or infected mice (B220/IL-10 cells, 15 days post-infection). **(C)** Phenotypic analysis of IL-10 producing B cells.

From the results described above, it can be concluded that trypomastigotes can induce functional regulatory abilities on B cells. The relevance of these findings was verified under *in vivo* conditions. Spleens from mice after two weeks of infection were collected and tested for the actual presence of IL-10-producing B cells (**Figure 5A**). A discrete population of regulatory IL-10-producing B cells was identified, thus providing further support to the previous observations (**Figure 5B**). Then, we analyzed the phenotype of IL-10-producing B cells by using three different panels of surface markers that consider their ontogeny and functional characteristics. The phenotype of these IL-10-producing B cells, a hallmark cytokine, was associated with a higher proportion of germinal center (Fas^+^/GL-7^+^), naïve or newly formed T1 B cells (NF-B, with a CD21^-^/CD23^-^ phenotype) and CD5^+^ regulatory B cell phenotypes (CD1d^+^/CD5^+^) (**Figure 5C**). Surprisingly, IL-10-producing B cells showed low expression of B220, an event characteristic of plasmablasts. Thus, confirming the results obtained in the *in vitro* model.

Therefore, *T. cruzi* trypomastigotes induced regulatory populations of B cells *in vitro* able to dampen the T cell response. In this model, we found that trypomastigote-stimulated B cells secrete and enhance the production of regulatory cytokines and membrane ligands such as IL-6, IL-10, PD-L1 and FasL. Moreover, IL-10, FasL and PD-L1 were also detected in the course of *T. cruzi* infection. Furthermore, in this model, we detected the presence of IL-21-producing B cells which is a cytokine associated with the development of B regulatory cells.

## Discussion

Within the physiology of infection by *T. cruzi*, the relevance of B cells was circumscribed to the production of antibodies. Despite the humoral response as a whole is not sufficient to effectively eliminate the parasite (Acevedo et al., 2018), relevant antibodies are induced during the infection such as lytic antibodies directed against anti-α-gal epitopes, neutralizing the activity of *trans*-sialidase and TcDAF (Tambourgi et al., 1993; Almeida et al., 1994; Buschiazzo et al., 2012). On the other hand, there are defined parasite factors such as *trans*-sialidase, proline-racemase and glutamate dehydrogenase that can, directly or indirectly, induce cytokine secretion and proliferation in B cells (Reina-San-Martín et al., 2000; Gao et al., 2002; Montes et al., 2006).

Although B cell ability to secrete cytokines and to act as an antigen-presenting cell is well known, it was historically considered as a secondary role. During the last years, it demonstrated that B cells turned out to be more than producers of immunoglobulins. Effector B cells are capable of secreting IL-2, IL-4, IL-6, IL-12, IL-13, IL-15, GM-CSF, and IFNγ, important cytokines in regulating the assembly and functionality of the immune system (Lund, 2008; Shen and Fillatreau, 2015). Furthermore, it was demonstrated that in *T. cruzi* infection, B cells are able to produce IL-17 *via* the action of *trans-*sialidase (Bermejo et al., 2013). In models of autoimmune diseases, chronic inflammatory processes and cancer, there are multiple reports of regulatory B cells (Gorosito Serrán et al., 2015; Sarvaria et al., 2017). Regulatory B cells (Breg) are a heterogeneous population that produces IL-10, IL-35 and/or TGFβ capable of inhibiting the Th1, Th17 and CD8^+^ responses, as well as the secretion of pro-inflammatory cytokines such as IFNγ or TNFα by macrophages or dendritic cells (Catalán et al., 2021). Furthermore, they could favor the development of regulatory T cells that, in turn, might impede the resolution of the pathology (Burdin et al., 1997; Candando et al., 2014; Lykken et al., 2015; Rosser and Mauri, 2015; Catalán et al., 2021). Other molecules have been associated in the generation of Breg such as APRIL and Blimp-1 (Fehres et al., 2019; Wang et al., 2019). In **Figure 3**, we showed that in our model we could detect the up-regulation of APRIL transcript.

So far, multiple populations of Breg have been identified according to their surface markers and the cytokines they release. The reported B populations that can express IL-10 are T2-MZP (CD21^+^/CD23^+^), classical B10 (CD5^+^/CD1d^+^), MZ (CD21^+^/CD23^lo/-^), plasma cells and plasmablasts (CD138^+^) (Rosser and Mauri, 2015). At present, the induction mechanism of these cells has not been fully elucidated and there is no known specific transcription factor that regulates this cell differentiation which might reflect the plasticity of these cells. However, some required pathways have been described. In an inflammatory context (IL-1β, IL-2, IL-6 and IFNα, among others) there are at least two known pathways that are involved in the generation of these cells, the B receptor-dependent (BCR) and the Toll-like receptors dependent. In the first one, it would appear that signaling through the BCR is critical. In this pathway, the Bregs would be antigen-specific and when presenting the antigen in the context of MHC-II to the lymphocyte T CD4^+^ they would be stimulated by it *via* CD40/CD40L and IL-21. In the second route, it was possible to determined that the TLR2/4/9 receptors are involved and their signaling *via* MyD88 plus IL-21 would induce the secretion of IL-10 by B cells (Yoshizaki et al., 2012; Horikawa et al., 2013; Lykken et al., 2015; Okada et al., 2018).

The presence of Breg cells and their regulatory activity on the T cell response have recently been described in the course of infectious diseases caused by pathogens of the genera *Plasmodium*, *Listeria*, *Leishmania*, and *Schistosoma* (Horikawa et al., 2013; Gorosito Serrán et al., 2015; Silva-Barrios et al., 2017). Within the parasitic infections, the B populations capable of developing this regulatory profile differ in each case and in several of them they were little studied. For example, in *Leishmania donovani* a CD19^+^/CD21^+^/CD23^lo^ (marginal zone phenotype) secreting IL-10 population was identified, while in the related *Leishmania major*, the identified population was CD19^+^/CD1d^+^/CD5^lo^ (B10 phenotype) (Ronet et al., 2010). On the other hand, in *Plasmodium* they are described as IL-10 secreting B220^+^ and in *Schistosoma* as IL-10 and TGF-β secreting CD19^+^/CD1d^+^ cells (Liu et al., 2013; Van Der Vlugt et al., 2014).

In *T. cruzi* infection, previous works with B cell-depleted Xid or µMT mice provided evidence supporting that B cells can regulate the T cell response (Minoprio et al., 1993; Gorosito Serrán et al., 2017; Fiocca Vernengo et al., 2020). However, the mechanisms and cell populations involved in the T CD4^+^ modulation process are still not understood.

In this study, we demonstrated that the infectious form of the parasite directly stimulated B cells that can regulate T CD4^+^ cells, impairing their proliferation and inducing an apoptotic process.

Notably, we determined that trypomastigotes directly induced naïve B cell proliferation accompanied by secretion of IL-6 and IL-10. These molecules are involved in B activation and cell proliferation. Although here we have shown that they are not involved in B cell division, both regulated the immunoglobulin secretion. There are several reports that support the induction of IL-6 and IL-10 *via* TLR4 (Greenhill et al., 2011; Sanin et al., 2015). It has also been reported that *T. cruzi* GIPLs are able to stimulate TLR4 (DosReis et al., 2002). Since B cells express TLR4 (Ma et al., 2015), the mechanism of induction of these cytokines might be explained in this way. Also, we detected the presence of APRIL and BAFF transcripts which are classically expressed by myeloid accessory cells, representing a hypothetic autocrine mechanism induced directly by trypomastigotes on B cells in the context of CD40 stimulation. Even these growth factors are canonically secreted by another cell source rather than B cells, evidence has emerged of its expression by B cells themselves both *in vitro* and *in vivo* (Mackay et al., 2003; Ng et al., 2005; Chu et al., 2007). Several reports state that toll-like receptors (TLRs) signaling could stimulate BAFF and APRIL expression in B cells and other antigen presenting cells (APCs) (Chu et al., 2007; Sakai and Akkoyunlu, 2017). In this line, pathogen-associated molecular patterns (PAMPs) from trypomastigotes have been identified, including GPI anchored mucins, membrane GIPL, DNA CpGs and parasite RNA (Rodrigues et al., 2012). Since B cells express variety and quantity of TLRs (Ma et al., 2015), the expression of BAFF and APRIL might be associated with their presence.

Bermejo et al. (2011) have reported that, in the context of *T. cruzi* infection, BAFF levels are elevated and it is related to polyclonal B cell response and antibody titers. Moreover, in another report of the same workgroup they observed that the *T. cruzi* antigen glutamate dehydrogenase induced B cells proliferation mediated by BAFF produced by CD11b^+^ cells. The authors hypothesized that these cells were macrophages but exist the possibility that they could be also CD11b^+^ expressing B cells (Montes et al., 2006).

In humans, circulating regulatory B10 results altered during the chronic stage and is associated with improved cardiac function in the indeterminate stage of Chagas disease (Passos et al., 2019; Girard et al., 2021). Also, since we observed a regulatory phenotype on parasite-stimulated B cells we tested for the presence of IL-21, a cytokine related to regulatory B10 cell generation (Horikawa et al., 2013). Surprisingly, given that IL-21 is paradigmatically mainly produced by CD4^+^ cells, we have found IL-21 expression in B cells during the experimental infection. This finding is noteworthy as IL-21 expression has previously been reported only in Hodgkin’s lymphoma (B-cell-related lineage) (Scheeren et al., 2008). It has been proved that IL-6 is able to induce the production of IL-21 in CD4^+^ cells (Dienz et al., 2009). It is also known that B cells are responsive to IL-6 and constitute a growth and differentiation factor. In our model, we showed that this cytokine is involved in IgG secretion (**Figure 2B**). Furthermore, NFAT, a downstream signaling molecule shared in the TCR and BCR cascade, has been shown to be involved in IL-21 secretion (Antony et al., 2004). Therefore, the combination of IL-6 and BCR signals in B cells could be plausible stimuli to induce IL-21 in these cells. Overall, the expression of B cell growth factors such as BAFF and APRIL plus IL-21 by B cells could represent a mechanism of *T. cruzi* to generate B-cell subsets that regulate the immune response against the pathogen.

We also showed that *T. cruzi* stimulated B cells damped T cell proliferation, the typically Th1-like cytokine secretion, and increased the apoptotic rate (Annexin V^+^/PI^-^) of CD4^+^ activated T cells. Moreover, recent evidence from Gorosito-Serran et al. (2015) described that the absence of B cells results in an uncontrolled CD4^+^ inflammatory response. These results suggest that *T. cruzi* trypomastigotes are able to manipulate B cells to restrain the CD4^+^ T cell activation inhibiting cell proliferation and increasing apoptosis induction.

The stimulation of CD40 on B cells was necessary for most of the described features or the enhancement of B regulatory cells generation in this model. This opens two hypotheses about the CD40L source *in vivo* during *T. cruzi* infection: the first, according to the paradigm, is that CD4^+^ cells present in lymphoid tissues are the natural CD40L source. Upon stimulation with CD40L from these cells, regulatory B cells are generated and constitute a restraint of the immune response against the parasite, contributing to the prevention of pathogen clearance from the host. The other possibility is that CD40L stimuli proceed from another source, like platelets. It is well-known that in the course of an acute infection with *T. cruzi*, platelet blood count decreases dramatically generating thrombocytopenia (de Titto and Araujo, 1988). In a previous work from our laboratory, we demonstrated that *trans-*sialidase from the parasite alters platelet surface glycan profile and induces their removal from circulation, promoting the marked platelet count fall (Tribulatti et al., 2005). In addition to this phenomenon, platelet activation results in its own disassembling into smaller fragments (vesiculation) and can also cause thrombocytopenia (Heemskerk et al., 2002; Taus et al., 2019). During platelet activation, they release several components, including CD40L. Some reports claim that platelet-derived CD40L might represent a relevant source of the circulating form, which is crucial for the proper development of the B response (André et al., 2002; Viallard et al., 2002; Sprague et al., 2008; Aloui et al., 2014). Moreover, the injection of small molecules that mimic the functional trimeric CD40L can control parasitemia and mortality (Fournel et al., 2005; Habib et al., 2007). Further research needed to be performed to clarify these issues.

In the context of *T. cruzi* infection we have found that IL-10 production by B cells is elicited by different subpopulations. Surprisingly, we detected that follicular phenotype B cells (Fas^+^/GL7^+^) express IL-10 upon *T. cruzi* infection. A recent report of *Leishmania amazonensis* infection also concludes that B2 follicular cells are relevant on IL-10 production, indicating that both pathogens might share common mechanisms relying on their phylogenetic similarity (Firmino-Cruz et al., 2018). Also, we observed that newly formed B cells (NF-B) constitute an important source of IL-10 in *T. cruzi* infection (**Figure 5**). It has been reported that NF-B are significant IL-10 producers in healthy individuals and expand in several viral (HIV, HCV) and bacterial infections, thus, they might represent a new regulating subset in parasitic infections, as Chagas disease (Giltiay et al., 2019). In addition, we observed the appearance of CD5^+^/CD1d IL-10^+^ B cells after infection according to several previous reports in different infectious processes, (Gorosito Serrán et al., 2015). This former phenotype is associated with the classical regulatory B cell subset, which shares molecular markers with B1 cells.

According to our *in vitro* results, the proliferation of purified murine B cells is induced by trypomastigotes *per se*, but a significant fraction of cells does not divide. However, upon CD40 stimulation the fraction of undivided cells was reduced. This fact reflects itself the heterogeneity of natural B subsets, each needing a different nature and combination of stimuli including TLR, BCR and CD40 among others. Moreover, it is well-known that *T. cruzi* induces a massive expansion of the B cell compartment, reflected in the characteristic splenomegaly, and a consequent polyclonal response. According to these facts, it seems reasonable that the expression of IL-10 by B cells is not confined to a unique subset but comes from different phenotypes with different ontogenesis.

All the evidence described here suggests that *T. cruzi* trypomastigotes are able to modulate the fate of B cells. In this condition, the stimulated B cells secrete various cytokines and immunoglobulins and module the response of T CD4^+^ cells by altering cytokine secretion, arresting cell division, and apoptosis induction. Our results add strong evidence of the central relevance of B cells during the pathogenesis of Chagas disease beyond the antibodies production.

## Data Availability Statement

The original contributions presented in the study are included in the article/**Supplementary Material**. Further inquiries can be directed to the corresponding author.

## Ethics Statement

The animal study was reviewed and approved by Committee on the Ethics of Animal Experiments of the Universidad Nacional de San Martín (UNSAM).

## Author Contributions

MS, RV, OC, and JM were responsible for the study design and/or funding acquisition. MS, AB, CAP, and JM performing the experiments. MS, OC, and JM were responsible for results analysis. Project supervision was performed by OC and JM. MS, OC, and JM were responsible for writing the original manuscript draft. All authors contributed to the article and approved the submitted version.

## Funding

This work was funded by grants from the Agencia Nacional de Promoción de la Investigación, el Desarrollo Tecnológico y la Innovación (ANPCyT), Argentina and from the National Institutes of Health, USA (grant R01AI104531 to OC). RV is supported by R01GM121595 from the National Institute of General Medical Sciences (NIH/NIGMS), 1R01CA233945 and from the National Cancer Institute of the National Institutes of Health (NIH/NCI) and the National Cancer Institute of the National Institutes of Health under Award Number P30CA240139. The funders had no role in the design of studies, data collection, and analysis, or in the decision to publish or the preparation of the manuscript.

## Author Disclaimer

This content is solely the responsibility of the authors and does not necessarily represent the official views of the National Institutes of Health.

## Conflict of Interest

The authors declare that the research was conducted in the absence of any commercial or financial relationships that could be construed as a potential conflict of interest.

## Publisher’s Note

All claims expressed in this article are solely those of the authors and do not necessarily represent those of their affiliated organizations, or those of the publisher, the editors and the reviewers. Any product that may be evaluated in this article, or claim that may be made by its manufacturer, is not guaranteed or endorsed by the publisher.
