# Supplementary material for: Trypanosoma cruzi Induces B Cells That Regulate the CD4+ T Cell Response
Source: Front Cell Infect Microbiol. 2022 Jan 5;11:789373. doi: 10.3389/fcimb.2021.789373 (PMC8766854; doi:10.3389/fcimb.2021.789373)
Supplement: Supplementary file 1 [file DataSheet_1.pdf]

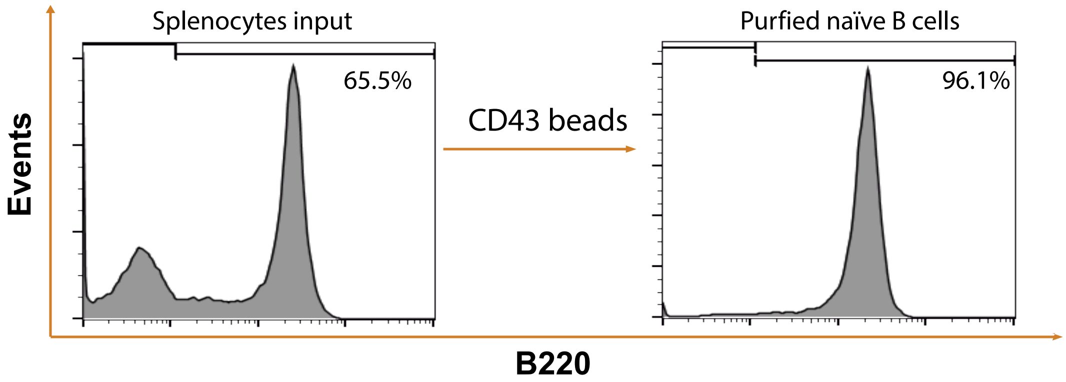

Supplemetary figure 1. Magnetic beads B cell purification. Dynabeads $CD43^{+}$ untouched B cells kit was used. Purification was measured by FACS using B220 B cell marker.
