# Supplementary material for: Trypanosoma cruzi Induces B Cells That Regulate the CD4+ T Cell Response
Source: Front Cell Infect Microbiol. 2022 Jan 5;11:789373. doi: 10.3389/fcimb.2021.789373 (PMC8766854; doi:10.3389/fcimb.2021.789373)
Supplement: Supplementary file 2 [file DataSheet_2.pdf]

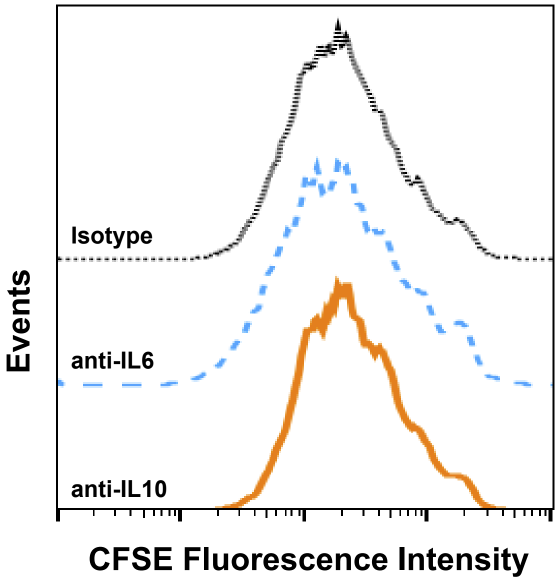

Figure Supplementary 2. Trypomastigote induced proliferation is IL-6 and IL-10 independent. Naïve B cells stained with CFSE were cocultured with trypomastigotes $1x10^6$ in the presence of antibodies able to block IL-6 and -10. Note the absence of modifications in the proliferative ability, as detected by flow cytometry.
