# Supplementary material for: Trypanosoma cruzi Induces B Cells That Regulate the CD4+ T Cell Response
Source: Front Cell Infect Microbiol. 2022 Jan 5;11:789373. doi: 10.3389/fcimb.2021.789373 (PMC8766854; doi:10.3389/fcimb.2021.789373)
Supplement: Supplementary file 3 [file DataSheet_3.pdf]

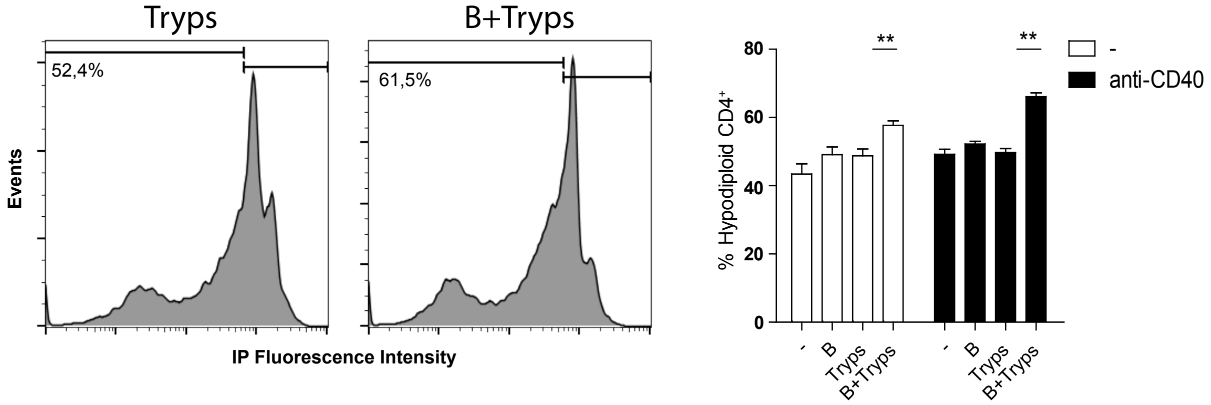

Figure Supplementary 3. Conditioned media from trypomastigote and B cells cocultures induced hypodiploidy in $CD4^+$ cells. $CD4^+$ cells were cultured in conditioned media from different conditions. -: none, B: B cells alone; Tryps: trypomastigotes; B+Tryps: B cells and trypomastigotes cocultures. Statistical analysis was performed using two-way ANOVA with Bonferroni correction. **p>0.001
